# Supplementary material for: Complexome profiling of the Chlamydomonas psb28 mutant reveals TEF5 as an early PSII assembly factor
Source: Plant Cell. 2025 Mar 17;37(6):koaf055. doi: 10.1093/plcell/koaf055 (PMC12164590; doi:10.1093/plcell/koaf055)
Supplement: koaf055_Supplementary_Data [file koaf055_supplementary_data.zip › psb28_interactive.zip!/plots/intensity/qualityPlots/peptides/_Cre02.g081250.t1.2.html]

### Cre02.g081250.t1.2

Trivial name:   
SeqCoverage [median]: 38.80  
Mass: 78.42 kDa  
  
 

| Sequence | MissedCleavage | Mass [Da] | wtIntensity | muIntensity | Score |
| --- | --- | --- | --- | --- | --- |
| AAKAEEPKQVEEPAPAK | 2 | 1791.94 | 615.98 | 0.00 | 30.11 |
| AAPPPAPEPVKR | 1 | 1228.69 | 2425.29 | 2051.14 | 63.73 |
| AEEPKQVEEPAPAK | 1 | 1521.77 | 0.00 | 521.02 | 92.94 |
| AEVEEVVVEEAPK | 0 | 1426.72 | 1885.31 | 3460.64 | 127.83 |
| AGSGFNFFGTGK | 0 | 1188.56 | 0.00 | 2726.33 | 86.90 |
| AKQAAEPKEEAQPEPEEAPAPK | 2 | 2344.15 | 438.95 | 0.00 | 27.90 |
| AQAASVVAELLK | 0 | 1198.69 | 993.76 | 0.00 | 115.57 |
| EAPKEEEEEAPAPK | 1 | 1552.73 | 332.56 | 0.00 | 57.14 |
| EAPKKEEPKEEEEAPAPK | 3 | 2035.01 | 646.51 | 647.70 | 48.90 |
| EAPKVEEEEAPAPK | 1 | 1522.75 | 1109.60 | 787.21 | 62.04 |
| EAPKVEEEEEAPAPK | 1 | 1651.79 | 430.55 | 0.00 | 50.16 |
| KEEPKEEEEAPAPK | 2 | 1609.78 | 0.00 | 1019.69 | 53.49 |
| KGTGFSALFATPK | 1 | 1323.72 | 260.71 | 1335.75 | 46.48 |
| KGTGFSFFGTGK | 1 | 1232.62 | 0.00 | 1517.55 | 48.66 |
| KTGTGFSFAFGAPK | 1 | 1414.72 | 0.00 | 1875.56 | 62.04 |
| LISLLQLEEDDAAVK | 0 | 1655.90 | 2099.98 | 0.00 | 157.68 |
| PAATAPGQPIASLSK | 0 | 1407.77 | 0.00 | 402.65 | 67.39 |
| QLADSGKPYAIVR | 1 | 1416.77 | 2058.86 | 564.41 | 79.49 |
| STESAPTYVPGTR | 0 | 1364.66 | 1643.10 | 0.00 | 104.79 |
| TGSGFSFFGTGK | 0 | 1191.56 | 715.23 | 2003.82 | 107.06 |
| VKEPEPEEEPAPK | 1 | 1477.73 | 424.26 | 0.00 | 57.14 |
| VVVVDGDTDGK | 0 | 1102.55 | 1107.78 | 1011.27 | 76.06 |
| VVVVDGDTDGKSK | 1 | 1317.68 | 6754.73 | 3892.86 | 77.19 |
|  |  |  | 23943.2 | 23817.6 |  |
